# Predictive capacity of a miRNA panel in identifying teratoma in post‐chemotherapy consolidation surgeries

**DOI:** 10.1002/bco2.143

**Published:** 2022-07-05

**Authors:** Joseph A. Moore, Michael J. Lehner, Simone Anfossi, Saumil Datar, Rebecca S. Tidwell, Matthew Campbell, Amishi Y. Shah, John F. Ward, Jose A. Karam, Christopher G. Wood, Lois L. Pisters, George A. Calin, Shi‐Ming Tu

**Affiliations:** ^1^ Department of Genitourinary Medical Oncology Division of Cancer Medicine, University of Texas MD Anderson Cancer Center Houston TX; ^2^ Department of Internal Medicine University of Texas Health Science Center at Houston Houston TX USA; ^3^ Department of Translational Molecular Pathology Division of Pathology/Lab Medicine, University of Texas MD Anderson Cancer Center Houston TX; ^4^ Department of Biostatistics University of Texas MD Anderson Cancer CenterTX Houston TX; ^5^ Department of Urology Division of Surgery, University of Texas MD Anderson Cancer Center Houston TX

**Keywords:** biomarker, micro RNA, miRNA, non‐seminomatous germ cell tumour, teratoma

## Abstract

**Objectives:**

To investigate the utility of a novel serum miRNA biomarker panel to distinguish teratoma from nonmalignant necrotic/fibrotic tissues or nonviable tumours in patients with NSGCT undergoing post‐chemotherapy consolidation surgery.

**Patients and methods:**

We prospectively collected pre‐surgical serum samples from 22 consecutive testicular NSGCT patients with residual NSGCT after chemotherapy undergoing post‐chemotherapy consolidation surgery. We measured serum miRNA expression of four microRNAs (miRNA‐375, miRNA‐200a‐3p, miRNA‐200a‐5p and miRNA‐200b‐3p) and compared with pathologic findings at time of surgery. Receiver operating characteristic (ROC) curves were performed to assess the ability of these miRNA to differentiate between teratoma and necrosis or viable malignancy.

**Results:**

Twenty‐two patients with NSGCT were split into two groups based on pathology at time of post‐chemotherapy consolidation surgery (teratoma group vs. necrosis/fibrosis/viable tumour group, i.e., NFVT). Patients with teratoma were older at diagnosis compared with those patients with NFVT (median age 28.7 vs. 23.9). Patients with NFVT were more likely to have embryonal carcinoma in their primary tumour (81.8% vs. 27.3%; *p* = 0.01). The majority of patients in both groups were stage III (63.6% vs. 72.7%). In this analysis, none of the miRNAs had good sensitivity or specificity to predict teratoma. There was no significant association between the expression levels of the miRNAs and the presence of teratoma. There was no statistically significant correlation between any of the miRNAs and teratoma size.

**Conclusion:**

This novel miRNA panel (miRNA‐375, miRNA‐200a‐3p, miRNA‐200a‐5p and miRNA‐200b‐3p) did not distinguish teratoma from nonmalignant necrotic/fibrotic tissues or nonviable tumours in patients with NSGCT undergoing post‐chemotherapy consolidation surgery.

## 1 INTRODUCTION

Traditional serum tumour markers alpha‐fetoprotein (AFP), beta‐human chorionic gonadotropin (β‐hCG) and lactate dehydrogenase (LDH) play an integral role in the diagnosis and management of testicular germ cell tumours (TGCTs). However, one major drawback of the current panel of serum tumour markers is their low sensitivity and specificity, especially in detecting residual tumour in patients undergoing post‐chemotherapy consolidation surgery. There is an unmet need for biomarkers to identify potentially lethal GCT phenotypes, such as teratoma, and to allow risk stratification of patients to avoid unnecessary treatment. One proposed type of biomarkers are microRNAs (miRNAs), which emerged as promising serum markers in testicular cancer and may predict viable tumour. Several recent studies have identified miR‐371a‐3p as a potential serum biomarker in TGCT with excellent sensitivity (>80%) and specificity (>90%), outperforming the classic serum tumour markers of GCTs.^1^, ^2^, ^3^, ^4^, ^5^, ^6^ If this stands true, miRNAs may allow for personalised treatment approaches to increase the number of patients who can achieve excellent clinical outcomes. Previous reports are mixed with regard to miRNA‐375 as a potential biomarker for teratoma.^7^, ^8^, ^9^, ^10^, ^11^, ^12^ The purpose of this study is to investigate the utility of a novel serum miRNA biomarker panel to distinguish teratoma from nonmalignant necrotic/fibrotic tissues or nonviable tumours in patients with NSGCT undergoing post‐chemotherapy consolidation surgery.

## 2 PATIENTS AND METHODS

We prospectively collected pre‐surgical serum samples from 22 consecutive TGCT patients with radiographic residual non‐seminomatous TGCT after chemotherapy undergoing post‐chemotherapy consolidation surgery (mostly post‐chemotherapy retroperitoneal lymph node dissections, PC‐RPLND) at MD Anderson Cancer Center in Houston, Texas, according to IRB‐approved protocols. Serum samples were obtained within 3 months prior to surgery. Patient #1 provided two preoperative samples; patient #5 underwent two separate post‐chemotherapy surgeries and thus had two preoperative serum samples; patient #6 provided one post‐operative sample in addition to his preoperative sample. We collected 1 mL of plasma (EDTA or citrate, non‐heparinized), 250 μL of which we used to measure miRNA in the plasma and 750 μL of which we used to measure miRNA in the exosomes, according to the methods used by an author (Dr. George Calin) in his laboratory at the MD Anderson Non‐coding RNA Center.^13^, ^14^ We performed RNA isolation from 100 μL of stored plasma collected on citrate using the Norgen kit (Norgen Biotek, Canada).

We measured miRNA expression of four microRNAs (miRNA‐375‐3p, miRNA‐200a‐3p, miRNA‐200a‐5p and miRNA‐200b‐3p) using the TaqMan miRNA quantitative reverse transcription polymerase chain reaction (qRT‐PCR) method (Applied Biosystems, Houston, TX), using a CFX384 real‐time PCR detection system (Biorad, Hercules, CA). Briefly, 10 ng of total RNA was reverse transcribed using the miRNA reverse transcription kit (Applied Biosystems) and a specific reverse‐transcription stem‐loop primer, according to the manufacturer's protocol. We ran all reactions in duplicate. The expression of a miRNA relative to the endogenous control was determined using the 2‐ΔCt method. If expression values for the endogenous control or for a specific miRNA were not obtained after 35 cycles of amplification in two successive experiments in duplicate wells, then the specific values were considered unavailable. Serum expression was validated and quantified by qRT‐PCR.

The expression of the various miRNA is presented as median with interquartile ranges (IQRs). Receiver operating characteristic (ROC) curves were performed to assess the ability of these miRNA to differentiate between teratoma and necrosis or viable malignancy. By comparing serum miR‐375‐3p levels before surgery with pathologic findings after surgery, we are able to correlate and validate testable biochemical measurements with definitive pathologic data. The miR‐375 expression was correlated with the residual teratoma burden.

Patient and tumour characteristics are tabulated for patients with and without teratoma. Selected differences between patients with versus without teratoma were compared with Fisher's Exact test using StatXact11 (Cytel Inc.). The levels of four miRs measured prior to surgery were modelled to predict the presence of teratoma with logistic regression. The associated ROC curves and area under that curve are presented. The odds ratios are presented for a change in unit of 0.0001. The association of the miRs and teratoma size is presented with scatter plots and Spearman's rank correlation. Patients with teratoma size ‘<0.5 cm’ were specified as 0.25 cm for correlation. Only univariate analyses are attempted given the small sample size. These analyses were performed in SAS 9.4 (SAS Institute, Inc. Cary, NC).

## 3 RESULTS

Our study cohort consisted of 22 patients with non‐seminomatous TGCT, which we split into two groups based on pathology at time of post‐chemotherapy consolidation surgery (teratoma group vs. necrosis /fibrosis/viable tumour group, i.e., NFVT). Baseline patient and tumour characteristics are shown in Table 1. Patients with teratoma were older at diagnosis compared with those patients with NFVT (median age 28.7 vs. 23.9). Patients with teratoma were also more likely to have teratoma in their primary tumour (72.7% vs. 18.2%; *p* = 0.01). Patients with NFVT were more likely to have embryonal carcinoma in their primary tumour (81.8% vs. 27.3%; *p* = 0.01). The majority of patients in both groups were stage III (63.6% vs. 72.7%). Patients with teratoma were less likely to be poor risk (27.3% vs. 45.4%). Patients with teratoma were more likely to have elevation of traditional serum tumour markers at baseline compared with patients with NFVT (90.9% vs. 70.0%). After chemotherapy and prior to surgery, 36.4% and 27.3% of patients had persistent elevation of traditional serum tumour markers in the teratoma (two LDH, one HCG, one AFP/LDH) and NFVT group (one LDH, one AFP, one HCG), respectively. In the NFVT group, residual tumour elements were infrequently observed. Two samples (18.2%) had embryonal carcinoma and one sample (9.1%) seminoma present. In the teratoma group, one patient (9.1%) had both embryonal carcinoma and yolk sac tumour present, whereas another patient (9.1%) had somatic transformation to carcinoma.

**TABLE 1:** Baseline patient characteristics

Parameter	Teratoma	Necrosis/fibrosis/viable tumour
Number of patients, *n* (%)	11 (50%)	11 (50%)
Age at diagnosis in years		
Mean (median)	30.3 (28.7)	26.5 (23.9)
Range	20–46	17–42
Age at PC‐RPLND in years		
Mean (median)	30.8 (29.1)	27.1 (24.1)
Range	20–46	18–42
Clinical stage at diagnosis, *n* (%)		
CSI	0	1 (9.1)
CSII	4 (36.4)	2 (18.2)
CSIII	7 (63.6)	8 (72.7)
IGCCCG prognosis group, *n* (%)		
Good	5 (45.4)	4 (36.4)
Intermediate	3 (27.3)	2 (18.2)
Poor	3 (27.3)	5 (45.4)
Tumour elements present in primary tumours, *n* (%)		
Burnt out primary	1 (9.1)	0
Choriocarcinoma	1 (9.1)	1 (9.1)
Embryonal carcinoma	3 (27.3)	9 (81.8)
Extragonadal germ cell tumour	1 (9.1)	2 (18.2)
GCNIS	1 (9.1)	0
Seminoma	3 (27.3)	5 (45.4)
Teratoma	8 (72.7)	2 (18.2)
Yolk sac tumour	1 (9.1)	1 (9.1)
Serum tumour markers pre‐chemotherapy, median (range)		
AFP (ng/mL; standard value < 8)	227 (1.5–631)	2.7 (1.4–70 160)
hCG (mIU/mL; standard value < 0.9)	132 (0.1–24 000)	8.5 (0.1–2086)
LDH (U/L; standard value < 224)	294 (142–1800)	628 (183–2552)
Serum tumour markers post‐chemotherapy, median (range)		
AFP (ng/mL; standard value < 8)	2.7 (1.2–92)	2.2 (1.3–37.4)
hCG (mIU/mL; standard value < 0.9)	0.1 (0.1–4.6)	0.1 (0.1–1.8)
LDH (U/L; standard value < 224)	190 (166–286)	191 (163–231)
Elevation in any serum tumour markers pre‐chemotherapy	10 (90.9)	7 (70.0)*
Elevation in any serum tumour markers post‐chemotherapy	4 (36.4)	3 (27.3)
Tumour elements present in residual tumours, *n* (%)		
Choriocarcinoma	0	0
Embryonal carcinoma	1 (9.1)	2 (18.2)
Seminoma	0	1 (9.1)
Somatic transformation to carcinoma	1 (9.1)	0
Teratoma	11 (100.0)	0
Yolk sac tumour	1 (9.1)	0
miR (GEOMEAN 2^−DCt^), median (range)		
375	0.00025 (0.00007, 0.00340)	0.00016 (0.00004, 0.00057)
200a‐5p	0.00008 (0.00003, 0.00010)	0.00007 (0.00005, 0.00014)
200a‐3p	0.00003 (0.00001, 0.00036)	0.00002 (0.00001, 0.00003)
200b‐3p	0.00004 (0.00002, 0.00019)	0.00005 (0.00000, 0.00015)

Abbreviations: AFP, alpha‐fetoprotein; GCNIS, germ cell neoplasia in situ; hCG, human chorionic gonadotropin; LDH, lactate dehydrogenase; PC‐RPLND, post‐chemotherapy retroperitoneal lymph node dissections.

**FIGURE 1:**
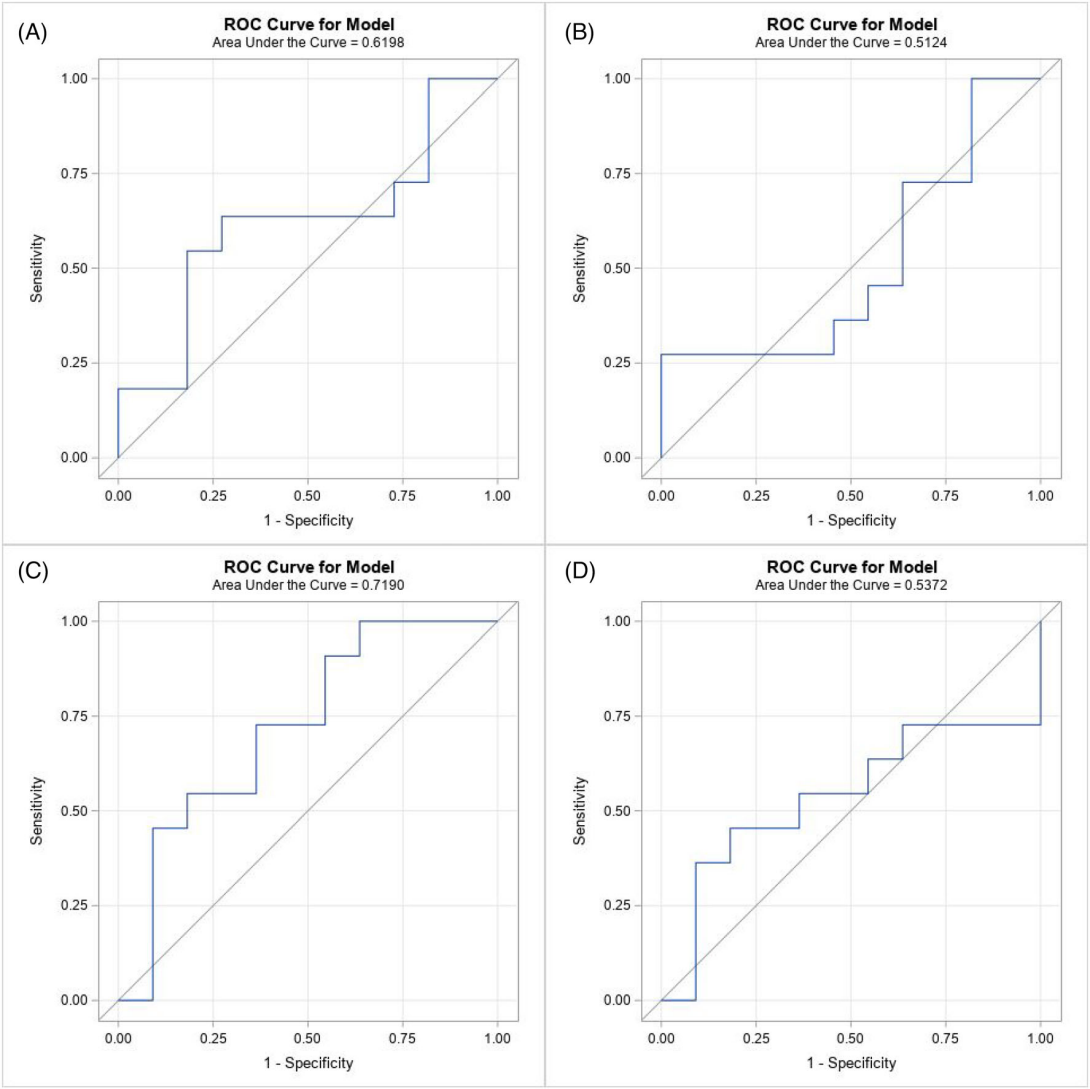
Receiver operating characteristic (ROC) curves predicting presence of teratoma by miRNA‐375 (A), 200a‐5p (B), 200a‐3p (C) and 200b‐3p (D)

**FIGURE 2:**
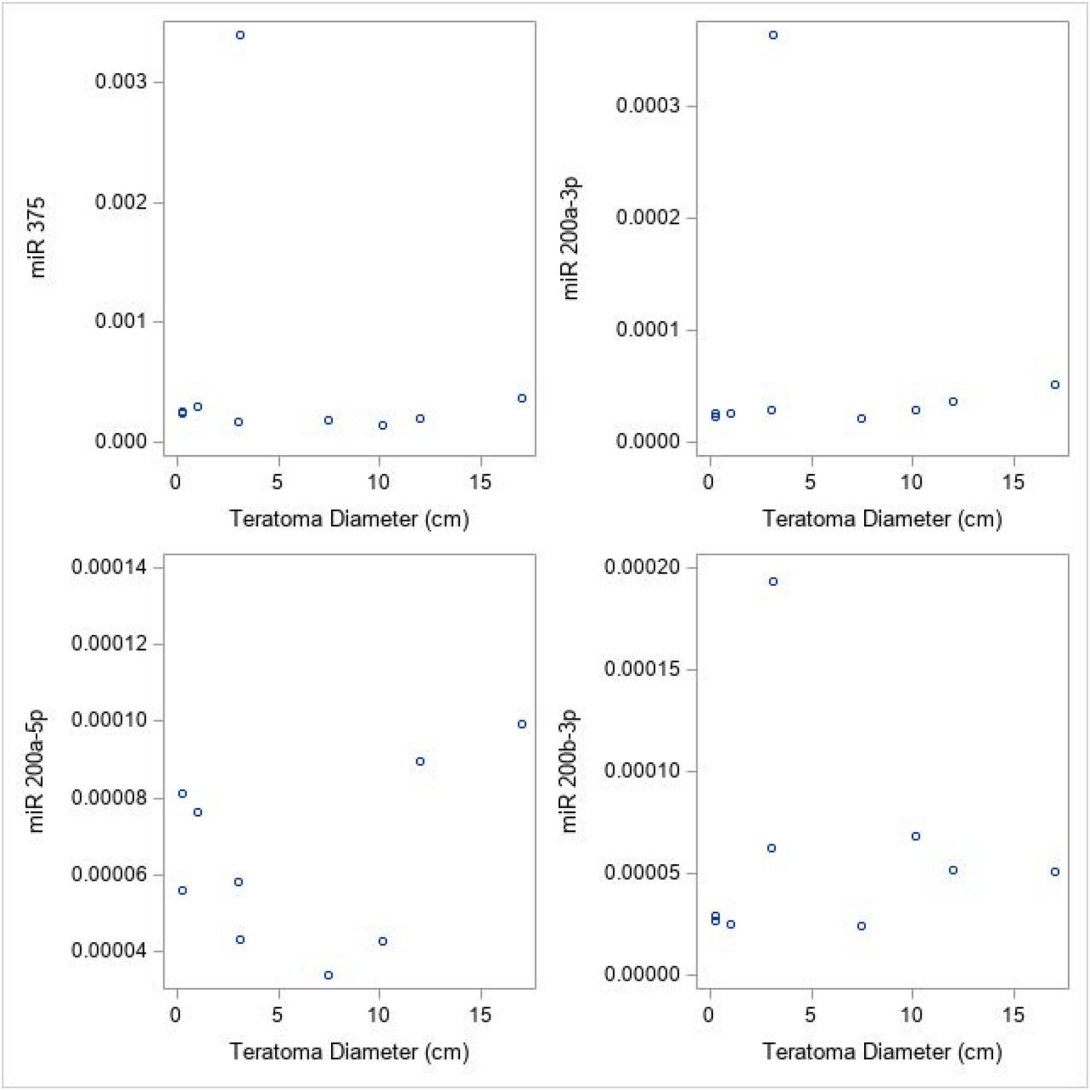
Each miRNA with teratoma diameter

Figure 1 presents the ROC curves measuring the ability of each miRNA to predict teratoma. In our analysis, none of the miRNAs have good sensitivity or specificity to predict teratoma. Table 2 presents the odds ratios for the presence of teratoma for each increase in miRNA normalisation of 0.0001. We found no significant association between the expression levels of the miRNAs and the presence of teratoma. Spearman's correlation was calculated in Table 3 in order to analyse whether teratoma size correlates with miRNA elevation. A total of 9 patients with teratoma had known maximum diameter, with 2 patients <0.5 cm. We did not find a statistically significant correlation between any of the miRNAs and teratoma size. Figure 2 presents the scatterplots for each miRNA with teratoma size.

**TABLE 2:** Odds ratio for presence of teratoma for every increase of 0.0001 in miRNA (GEOMEAN 2^−DCt^)

miRNA	Odds ratio (95% Confidence interval)	AUC	*p* value
375	0.90 (0.68, 1.19)	0.62	0.45
200a‐5p	3.29 (0.12, 88.75)	0.51	0.48
200a‐3p	0.00 (0.00, 14.04)	0.72	0.12
200b‐3p	1.10 (0.15, 7.86)	0.54	0.92

Abbreviation: AUC, area under the ROC curve.

**TABLE 3:** Spearman's correlation between teratoma size and miRNA (GEOMEAN 2^−DCt^) for *N* = 9

miRNA	Correlation with teratoma size	*p* value
375	−0.09	0.81
200a‐5p	0.16	0.68
200a‐3p	0.57	0.11
200b‐3p	0.33	0.39

## 4 DISCUSSION

GCTs have an overall cure rate of over 90%. Two ways to enhance the clinical outcome of a highly curable cancer are (1) to identify potentially lethal phenotypes and (2) to avoid unnecessary treatments and minimise treatment burden and morbidities. In theory, one can accomplish these goals by using specific biomarkers to match distinct tumour subtypes with the most appropriate and beneficial treatment modalities.

Currently, the standard of care to manage all residual masses >1 cm after chemotherapy in patients with normal serum tumour markers is PC‐RPLND to remove persistent lymph nodes that may contain mature teratoma in 30–40% or viable GCT in 10–20% of patients.^15^, ^16^, ^17^, ^18^, ^19^ However, not all residual tumours contain these pathologic entities; in fact, necrotic/fibrotic tumour and inflammatory tissue at the surgical site are quite common. Unfortunately, current imaging and available tumour biomarkers cannot differentiate teratoma from nonmalignant necrotic/fibrotic tissue or nonviable tumour. When surgery is particularly challenging and potentially treacherous (e.g., near a vital organ or structure; innumerable lesions in the lungs and liver), removing the residual malignant tumours is necessary and justifies the risks. However, removing nonmalignant tissues is completely unwarranted. Currently, the best option is to monitor and selectively remove lesions that grow or are biopsy‐positive (despite sampling errors) in such cases. Unfortunately, a delay in surgery for patients who harbour an indolent but refractory YST/teratoma and who are psychosocially challenged (e.g., noncompliant, no insurance) may entail a death sentence in an otherwise curable cancer.

miRNA regulates gene expression by base‐pairing with target mRNAs, and each miRNA can target dozens to hundreds of genes. As many as 30% of all genes are thought to be under miRNA control. miRNAs that specifically activate or repress traits such as pluripotency and self‐renewal that characterise stem cells may operate similarly in cancer stem cells.^20^ Therefore, miRNA expression profiles may provide a reliable way to classify tumour subtypes by accurately reflecting the developmental lineages of their malignant cells of origin.^21^ Advantages of using miRNAs as serum biomarkers include the ease of measurement by qRT‐PCR, presence in minimal amounts of serum and ability to use formalin fixed, paraffin‐embedded tissues instead of only fresh frozen samples.

Recently, Shen et al. used the cohort of patients with TGCTs from The Cancer Genome Atlas (TCGA) to identify molecular characteristics that classify TGCT types and showed that miR‐371a‐3p was dramatically overexpressed in seminoma, embryonal carcinoma and mixed non‐seminomatous TGCT but minimally expressed in teratoma.^7^ Conversely, miR‐375‐3p was highly expressed in teratoma, yolk sac tumour and mixed non‐seminomatous TGCT containing teratoma or yolk sac tumour but minimally expressed in embryonal carcinoma or seminoma.^7^ miR‐375 could be a useful biomarker to distinguish teratoma from necrotic/fibrotic tissue or nonviable tumour in residual masses following chemotherapy, which would help avoid unnecessary RPLNDs and other surgeries after chemotherapy.

Our study sought to test this hypothesis by measuring the expression level of miRNA‐375‐3p, along with three other biomarkers, miRNA‐200a‐3p, miRNA‐200a‐5p and miRNA‐200b‐3p. These four miRNA were chosen as they were four of the most differentially expressed miRNAs in teratoma according to the work by Shen et al.^7^ In addition, the miR‐200 family seems to play an important role in tumourigenesis, epithelial‐to‐mesenchymal transition and tumour cell migration.^22^ Our results demonstrate that there is no statistically significant correlation between any of the four miRNAs and residual teratoma. Interestingly, Patient 1 seemed to have elevated levels of miRNA‐375‐3p, miRNA‐200a‐3p and miRNA‐200b‐3p in the preoperative samples collected, and the final pathology was teratoma; however, this patient was an exception. Our study is limited by the small sample size, and therefore, caution must be taken when interpreting these results. However, given these results and the negative results of other groups, we see little utility in investigating these specific miRNAs further. Identifying a serum tumour marker with the ability to predict teratoma remains an unmet critical need.

## 5 CONCLUSION

This study explored the utility of a novel miRNA panel (miRNA‐375‐3p, miRNA‐200a‐3p, miRNA‐200a‐5p and miRNA‐200b‐3p) and found that this panel did not distinguish teratoma from nonmalignant necrotic/fibrotic tissues or nonviable tumours in patients with NSGCT undergoing post‐chemotherapy consolidation surgery.

## CONFLICT OF INTEREST

The authors declare that they have no conflicts of interest.

## AUTHOR CONTRIBUTIONS

JM, ML, SA, SD, GC and ST performed the conceptualization. JM, SA, RT, GC and ST performed the methodology. JM, SA, GC and ST did the formal analysis. JM, SA and ST did the data curation. JM, ML, SA, SD, RT and ST were responsible for the writing—original draft preparation; JM, ML, SA, SD, RT, MC, AS, JF, JK, CW, LP, GC and ST were responsible for the writing—review and editing. All authors have read and agreed to the published version of the manuscript. All of the authors meet criteria for authorship, have read the manuscript and have approved this submission.
